# Predictors of Drug Injection in High-Risk Populations of Prisoners with a History of Tattooing: A Cross-Sectional Study

**Published:** 2019-01-09

**Authors:** Saeede Jafari, Ghobad Moradi, Mohammad Mehdi Gouya, Fatemeh Azimian Zavareh, Ebrahim Ghaderi

**Affiliations:** ^1^ Student Research Committee, Kurdistan University of Medical Sciences, Sanandaj, Iran; ^2^ Social Determinants of Health Research Center, Research Institute for Health Development, Kurdistan University of Medical Sciences, Sanandaj, Iran; ^3^ Iranian Center for Communicable Diseases Control, Ministry of Health & Medical Education, Tehran, Iran

**Keywords:** Drug injection, Prisoners, Tattooing

## Abstract

**Background:** Transmitting blood-borne diseases is alarming in places with high prevalence of people who inject drugs. This study aimed to determine the prevalence of drug injection and its related predictors among prisoners with a history of tattooing in Iran.

**Study design:** Cross-sectional study.

**Methods:** By using a census sampling, 5493 prisoners with a history of tattooing of 11988 prisoners participated for hepatitis B and C bio-behavioral surveillance surveys (BSS) in prisons of Iran, during 2015-2016 from 55 prisons in 19 provinces were assessed. The data for the BSS were collected using face-to-face checklist-based interviews. Weighted prevalence and the association between variables and history of drug injection were determined using Chi-square test and adjusted odds ratio (AOR) was estimated through multivariate logistic regression test using survey package.

**Results:** The mean age of participants was 33.9 ± 8.3 yr. Most of them were male (96.4%) and had a history of drug use (85.4%). The prevalence of drug injection among drug users was 20.2%, of which 33.9% had a history of shared injection. The prevalence of drug injection among prisoners with a history of tattooing is associated with male gender (*P*=0.047), age ≥35 yr (*P*<0.001), being single (*P*=0.002), being divorced/widow (P=0.039), and a history of imprisonment (*P*<0.001).

**Conclusion:** The prevalence of drug injection increases in the presence of other high-risk behaviors. It is necessary to initiate harm reduction programs and preventive interventions in groups with multiple high-risk behaviors.

## Introduction

Today, unsafe, shared injections is one of the most important ways of transmitting blood-borne diseases (BBD)^1^. The risk of transmitting BBD is alarming in places with high prevalence of people who inject drugs (PWID). Prison is one of these places. The prevalence of PWID in prison varies from country to country but it was observed with the highest rates in Taiwan (62.8%)^2^ and in England (64%)^3^. In Iran, despite different estimations of prevalence in the provinces, this rate is reported to be 16% among all prisoners^4^.

The prevalence of BBD in prisons is associated with high-risk behaviors such as the history of drug injection ^5,6^, the history of tattooing ^7,8^, and homosexual sex^9^. Some individuals simultaneously have several high-risk behaviors. For example, the prevalence of tattooing is markedly high in prisoners who inject drugs ^10,11^. This can be justified by the almost identical nature of both high-risk behaviors which involve skin piercing and the insertion of an external object into the body by syringes. In these cases, the prevalence of BBD increases in individuals^12^.

The high prevalence of PWID in prisons with undesirable levels of sanitary facilities and health care leads to increased unsafe and shared injections^13^ and, ultimately, increased prevalence of BBD in this group^14^. In addition, the simultaneous presence of risk factors for BBD is contributing to the outbreak of disease in prisons and society due to the return of prisoners into the community.

The effective reduction of BBD in prisons is possible by reducing the associated risk factors. The behavioral surveillance surveys (BSS) are among the most reliable methods for tracking high-risk behaviors associated with BBD in high-risk groups such as prisoners^15^. Therefore, it is necessary to identify high-risk behaviors and the related predictors using the BSS to design appropriate and evidence-based interventions in order to increase health level in prisons and the community. In particular, there is no information on the drug injection and its related factors in key populations, such as prisoners with a history of tattooing.

We aimed to determine the prevalence of drug injection among prisoners with a history of tattooing using data from behavioral surveillance surveys in prisons during 2015-16.

## Methods

### Design of Study

This research is a cross-sectional descriptive-analytical study which is approved by the Ethics Committee of Kurdistan University of Medical Science with the code No. MUK.REC.1395.280.

### Participants and Sampling Method

The present study is part of a series of repetitive BSS of Iranian prisoners carried out in 2015 and 2016. The studied population includes 5493 prisoners with a history of tattooing. They were selected through census sampling among 11988 prisoners who had been participated and provided written informed consent form for hepatitis B and C BSS in prisons of Iran during 2015-2016 from 55 prisons in 19 provinces^7^.

### Data Collection Tool

The data for the national BSS were collected using face-to-face checklist-based interviews by trained interviewers. The checklist used in the national plan has 7 sections involving questions about demographic variables, non-sexual high-risk behaviors (history of drug injection, shared injection, piercing (Cupping or a process in which each pierce in whatever part of skin and mostly in soft parts of body like ear, nose, mouth, tongue, navel, and breast occurs by a needle for using jewelry) and tattooing), sexual high-risk behaviors (history of extramarital sex, number of heterosexual/homosexual partners and condom use), history of sexually transmitted infection (STI), and Hepatitis-related knowledge (including 6 questions toward ways of HBV/HCV transmission. Comprehensive knowledge was considered if the prisoner correctly recognized three ways of transmission). The checklist was constructed in 2009 based on international guidelines and protocol proposed for bio-behavioral surveys^4^.

### Statistical Analysis

The analyses were performed using Stata/SE 14.0 survey package. Chi-square test was used to estimate the weighted prevalence of drug injection and to determine its relationship with the qualitative variables. Weighting was based on the post-stratification method and the post-weight instrument, and the distribution frequency of post-strata was determined using information about the willingness of subjects to participate in the BSS. In addition, multivariate logistic regression was used to estimate the final model based on variables with *P*<0.2 in the univariate analysis and to calculate the adjusted odds ratio (AOR) as well as the 95% confidence interval (95% CI).

## Results

The mean age of participants in this study was 33.9 ± 8.3 yr. Most of the prisoners with a history of tattooing were male (96.4%), married (55.9%), non-academic education (97.1%), working (91.4%), and had a history of imprisonment (67.2%). In addition, a significant percentage of the participants were drug users (85.4%) and had a history of extramarital sex (67.5%). *P* values, the frequency distribution of demographic and behavioral variables is presented in Table 1.

**Table 1:** Frequency distribution of demographic and behavioral variables and weighted prevalence of drug injection in 5493 prisoners with a history of tattooing, 2015-2016

**Variables**	**Without drug** **injection**		**With drug** **injection**		***P*** ** value**
	**Number**	**Percent**	**Number**	**Percent**	
Gender					
Female	96	91.4	6	8.6	0.011
Male	3507	79.4	966	20.6	
Age (yr)					
<35	2102	82.8	447	17.2	0.001
≥35	1484	75.9	523	24.1	
Marital Status					
Single	1435	77.5	432	22.5	0.013
Divorced/ widow	331	75.3	126	24.7	
Married	1836	82.0	411	18.0	
Educational Level					
Illiterate – primary school	1346	80.4	359	19.6	0.321
Junior high school – Diploma	2164	79.1	593	20.9	
University	92	88.0	18	12.0	
Job Status					
Jobless	245	82.7	58	17.3	0.131
working	2988	79.5	821	20.5	
History of imprisonment in the recent 10 yr		
No	1154	89.9	141	10.1	0.001
Yes	2428	75.8	824	24.2	
Number of imprisonmentin the recent 10 yr		
1-2	1387	83.1	306	16.9	0.001
3-4	614	73.2	235	26.8	
³5	403	58.6	281	41.4	
Duration of imprisonment in the recent 10 yr			
≤5	1359	78.9	398	21.1	0.001
>5	342	63.9	207	36.1	
History of piercing in lifetime			
No	1812	81.1	431	18.9	0.327
Yes	1782	78.5	539	21.5	
History of extramarital sex in lifetime			
No	1022	84.1	204	15.9	0.050
Yes	2220	77.3	694	22.7	
Number of heterosexual/homosexual partners in extramarital sex	
1	358	81.4	88	18.5	0.377
2-3	1012	77.0	321	23.0	
>3	662	75.8	218	24.2	
Condom use in extramarital sex			
Never	472	75.4	154	24.6	0.217
Sometime	1267	76.6	437	23.4	
Always	452	80.8	95	19.2	
History of STI in the recent year			
No	3344	80.0	885	20.0	0.163
Yes	248	76.8	85	23.2	
Score of Hepatitis-related knowledge (out of 6)		
<4	1044	81.2	275	18.8	0.182
³4	1495	76.8	507	23.2	

The prevalence of drug injection among drug users was 20.2% (95% CI: 17.3, 23.5) and the prevalence of shared injection among PWID was 33.9% (95% CI: 27.2, 41.5). The prevalence of drug injection was significantly higher in men than in women (20.6% vs. 8.6%), in individuals with age 35 yr and over than those with age <35 yr (24.1% vs. 17.2%), in singles and divorced/widows than in married (22.5% and 24.7% respectively vs. 18%), in people with a history of imprisonment than in people without imprisonment record (24.2% vs. 10.1%), in prisoners with more than 5 times imprisonments than those with 1-2 times imprisonments (41.4% vs. 16.9%), and in inmates with a duration of imprisonment more than 5 yr than those with less than 5 yr (36.1% vs. 21.1%) (Table 1).

The results of multivariate logistic regression showed that the prevalence of drug injection among prisoners with a history of tattooing is associated with male gender (AOR: 3.02, 95% CI: 1.01, 9. 03), age≥35 yr (AOR: 1.89, 95% CI: 1.39, 2.56), being single (AOR: 1.62, 95% CI: 1.21, 2.18), being divorced/widow (AOR: 1.55, 95% CI: 1.02, 2.35), and a history of imprisonment (AOR: 2.70, 95% CI: 1.82, 4.00) (Table 2).

**Table 2:** Predictors of drug injection among 5493 prisoners with a history of tattooing, 2015-2016

**Variables**	**OR (95% CI)**	***P*** ** value**	**AOR (95% CI)** ^a^	***P*** ** value**
Gender				
Male vs. Female	2.74 (1.23, 6.09)	0.015	3.02 (1.01, 9.03)	0.047
Age (yr)				
≥35 vs. <35	1.53 (0.19, 1.96)	0.001	1.89 (1.39, 2.56)	0.001
Marital status				
Single vs. Married	1.33 (1.06, 1.66)	0.015	1.62 (1.21, 2.18)	0.002
Divorced/ widow vs. Married	1.50 (1.09, 2.05)	0.013	1.55 (1.02, 2.35)	0.039
Job status				
Jobless vs. Working	0.81 (0.62, 1.07)	0.131	1.02 (0.69, 1.50)	0.932
History of imprisonment in the recent 10 yr		
Yes vs. No	2.85 (2.18, 3.72)	0.001	2.70 (1.82, 4.00)	0.001
History of extramarital sex in lifetime		
Yes vs. No	1.55 (1.00, 2.42)	0.051	1.22 (0.73, 2.05)	0.443
History of STI in the recent year		
Yes vs. No	1.20 (0.92, 1.57)	0.163	1.35 (0.96, 1.91)	0.083
Score of hepatitis-related knowledge (out of 6)		
<4 vs. ³4	0.76 (0.51, 1.14)	0.182	0.84 (0.52, 1.34)	0.451

^a^ Adjusted odds ratio

It is notable that the variables of the history of imprisonment, number of incarceration, and duration of imprisonment that had criteria (p<0.2 in the univariate analysis) to entrance in to the final model had correlation with each other. So, the variable of the history of imprisonment was entered into multivariate logistic regression model because of it′s higher significant OR. ((OR: 2.85, 95% CI: 2.18, 3.72), (OR:1.86, 95% CI: 1.59, 2.17), (OR:2.11, 95% CI: 1.73, 2.58), respectively)

## Discussion

According to our study, the prevalence of drug injection among Iranian prisoners with a history of tattooing is 20.2%. Although there is no study on the prevalence of drug injection among prisoners with a history of tattooing, comparing this with the prevalence of drug injection among prisoners in neighboring countries of Iran (Pakistan 39%^16^ and Azerbaijan 33.7%^17^) showed that, despite the high prevalence of drug use in this group of individuals (85.7%), this rate is significantly lower for Iran. This may be due to differences in the pattern of drug use in Iran compared to other countries. However, comparing this rate with the prevalence of drug injection among Iranian prisoners (16%)^4^ indicates a high prevalence of drug injection among prisoners with a history of tattooing. This finding consistent with another results^8^, illustrated that the prevalence of a high-risk behavior increases if there are other high-risk behaviors. Therefore, people with multi high-risk behaviors should be in priority for implementing harm reduction programs.

The findings revealed being single and being divorced/widow are associated with the prevalence of drug injection. Furthermore, drug injection is more frequent behavior in individuals who had extramarital sex. Although this difference is not significant. This highlight the importance of marriage in the community. In fact, married people are committed to adhering to ethical principles and, therefore, the odds of high-risk behaviors are reduced in these individuals. Accordingly, the statesmen take steps toward easy marriage in the society. In addition, it is important because extramarital sex and drug injection are the main identified transmission ways of BBD all-around the world and Iran also. Therefore, existing both of them can increase odds of infected. Therefore, implementing harm reduction programs with priority in prisoners with a history of extramarital sex and drug injection simultaneously can facilitate the achievement of 2030 goal^18^.

The prevalence of drug injection is associated with age≥35 yr and male gender. Since male with age group of 35-39 yr olds have the highest employment rate, addiction and its complication in this economically active population can cause serious and irreparable damage to the country's economy. Consequently, in designing interventions and harm reduction programs it is necessary to paid attention to male over 35 yr old as a vulnerable group.

The prevalence of drug injection was associated with a history of imprisonment. In agreement with this finding, drug use in prison was associated with prolonged imprisonment^19^. The odds of exposure to high-risk behaviors increases in incarceration. It is possible because of jobless, more leisure time, and the presence of high-risk groups such as PWID and sexually active. Despite the fact that prison is a place for correcting and reforming individuals, it is high-risk environment. Grouping in prisons based on criminal records and duration of incarceration and increasing supervision and restriction measurements in prisons are recommended.

Data were related to the BSS and the sampling method was multi-stage sampling, an important part of which is cluster sampling, selection bias is one of the most important limitations of this study. Therefore, weighting in the analyses was used to resolve the limitation.

## Conclusion

The prevalence of drug injection is higher among prisoners with other high-risk behaviors and this rate is associated with male gender, age≥35 yr, being unmarried (single/ divorced/ widow), and history of imprisonment. Although some of these predictors may not be modifiable, they can be intervened. In this regard, grouping in prisons based on the criminal records and duration of imprisonment, providing appropriate training on high-risk behaviors and its complications resulting from it, implementing harm reduction programs for prisoners with a history of extramarital sex and drug injection, as well as facilitating marriage in the community are recommended. In addition, these people should be in priority for implementing harm reduction programs.

## Acknowledgements

The researchers appreciate the sincere help provided by the State Prisons and Security and Corrective Measures Organization, and the Center for Communicable Diseases Control at the Ministry of Health and Medical Education, as well as all the prisoners who participated in the study.

## Conflict of interest statement

The authors declare that there is no conflict of interests.

## Funding

This study was conducted under the financial support of the Deputy of Research and Technology of Kurdistan University of Medical Sciences.

## Highlights

- The prevalence of drug injection among prisoners with a history of tattooing is significantly higher than those without it. Therefore, initiating harm reduction programs and preventive interventions in groups with multiple high-risk behaviors are priority.
- The prevalence of shared injection among prisoners with multiple high-risk behaviors (injection drug use and history of tattooing) is remarkable.
- The odds of drug injection increase in prisoners with a history of incarceration. Therefore, grouping in prisons based on the criminal records and duration of imprisonment is necessary.
